# The Incidence of Healthcare-Associated Infections, Their Clinical Forms, and Microbiological Agents in Intensive Care Units in Southern Poland in a Multicentre Study from 2016 to 2019

**DOI:** 10.3390/ijerph18052238

**Published:** 2021-02-24

**Authors:** Elżbieta Rafa, Marta Z. Wałaszek, Michał J. Wałaszek, Adam Domański, Anna Różańska

**Affiliations:** 1 State Higher Vocational School in Nowy Sącz, 33-300 Kraków, Poland; raela@vp.pl; 2 State Higher Vocational School in Tarnów, St. Luke Provincial Hospital in Tarnów, 33-100 Tarnów, Poland; mz.walaszek@gmail.com; 3 Polish Society of Hospital Infections, 31-121 Kraków, Poland; michalj.walaszek@gmail.com; 4 Department of Distributed Systems and IT Equipment, Electronics and Computer Science, Faculty of Automatic Control, The Silesian Technical University, 44-100 Gliwice, Poland; Adam.Domanski@polsl.pl; 5 Chair of Microbiology, Jagiellonian University Medical College, Czysta str. 18, 31-121 Kraków, Poland

**Keywords:** healthcare-associated infections (HAI), intensive care unit (ICU), bloodstream infection, pneumonia, urinary tract infection, *Acinetobacter baumannii*, *Klebsiella pneumoniae*

## Abstract

Introduction. Healthcare-associated infections (HAIs) are a serious problem of modern medicine. Patients hospitalized in intensive care units (ICUs) develop HAI significantly more often than patients in other hospital units. Materials and Methods. Analysis involved HAIs from three ICUs in southern Poland. The study was conducted in 2016–2019 on the basis of methodology recommended by the Healthcare-Associated Infections Surveillance Network (HAI-Net) and European Centre for Disease Prevention and Control (ECDC). The objective was to analyse HAIs, their clinical forms, and microbiological agents. Results. The study included 3028 patients hospitalized for 26,558 person-days (pds) in ICU. A total of 540 HAIs were detected; incidence per 100 hospitalizations was 17.8%, incidence density per 1000 pds was 20.3. The mortality of patients with HAI was 16%, and in *Clostridioides* *difficile* infection (CDI), the mortality was 28%. The most common clinical form of HAI was bloodstream infection (BSI): 209 cases (incidence rate 6.9%), followed by pneumonia (PN): 131 (incidence rate 4.3%), and urinary tract infection (UTI): 110 cases (incidence rate 3.6%). The most frequently isolated bacteria were *Klebsiella pneumoniae* 16.4%, *Acinetobacter baumannii* 14.4%, *Staphylococcus aureus* 11.8%, and *Escherichia coli* 11.4%. Conclusions. A two-fold higher incidence rate of BSI was detected compared to the average incidence in European countries. BSI of unknown source (BSI-UNK) was predominant. *K. pneumoniae* and *A. baumannii* bacteria were the most often isolated microorganisms causing HAI. Infection control based on incidence rate for each type of infection is necessary in ICU to assess the epidemiological situation.

## 1. Introductions

Healthcare-associated infections (HAIs) are one of the most serious problems and challenges of modern medicine. HAIs lead to high mortality, prolonged hospital stay, and increased costs of therapy [1,2]. It is estimated that HAIs affect approximately four million people in the European Union each year, involving up to 10% of those hospitalized and responsible for up to 1% of the deaths of all patients admitted [3].

Antimicrobial resistance is one of the ten threats identified by the World Health Organization (WHO) and the one closely related to HAIs [4]. According to EARS-Net Reports of European Centre for Disease Prevention and Control (ECDC), Poland places among the countries with a substantial resistance of strains isolated from invasive infections [5]. Another ECDC report from the ESAC-Net indicates a higher rate of antibiotics consumption in the community sector in Poland than the average European rate. Although the analogous rate for the hospital sector is lower than the European average, some Polish studies dedicated to intensive care units (ICUs) have revealed troubling results of antibiotic consumption in these wards together with very high rates of resistant bacteria isolated from infections [6,7].

Basically, healthcare-associated infections and antimicrobial resistance are the areas of particular interest in infection control. HAIs occur more frequently in ICUs than in other hospital departments and many of them are life-threatening, despite the fact that these units are responsible for only 15–20% of all hospital beds [2]. The development of HAI can be facilitated by the general severe health condition in patients and their comorbidities; the risk is also increased when invasive devices are used, such as: vascular and urinary catheters, intubation, and the need for frequent direct contact between healthcare professionals and patients [1,8]. Around 20–50% of patients hospitalized in ICU develop HAI [1,9,10,11,12]. In the course of HAI treatment in ICU, microbiological diagnostic of infections is of considerable importance. Pinpointing the etiological agent of HAI results in the introduction of targeted therapy according to antibiogram, which may increase the therapeutic success. Additionally, each case of HAI in intensive care unit, especially caused by antimicrobial resistant pathogens, is connect with substantial financial consequences. Duszyńska et al. estimated the cost of HAIs in a Polish ICU to range from EUR 10,035 to 22,411, depending on the form of infections [13]. The economic burden, additionally to patient safety and clinical reasons, justify the active, targeted, and long-term surveillance of HAIs in ICUs. Implementation of a standardized system of HAI surveillance, monitoring of the occurrence of individual types of HAI, and reliable microbiological diagnostics of these infections may reduce the incidence of the most dangerous forms of HAI in ICU [14,15,16,17].

There is a lack of large multicentre studies, the results of which could be used to assess the epidemiological situation in Polish ICUs. Individual research papers concerning HAI in ICU are available in Poland. However, there are different approaches to HAI surveillance, and different recommendations prepared by some leading centres. Only within the HAI-Net project is there a possibility of choosing one of the two versions of protocol—standard or light. Standard protocol is based on detailed data gathered for all patients hospitalized for at least two days in ICUs, and light is based on the aggregated ward data with details only for infections [18]. Duszyńska et al. studied device-associated HAI in ICUs, based on the International Nosocomial Infection Control Consortium (INICC) and used the protocol and dataset similar to those recommended in the latest version of the standard HAI-Net ICU protocol by the ECDC [13]. Another multimodal surveillance of HAIs was implemented in an Italian intensive care unit of a large teaching hospital and described by Migliara G et al. [19]. Approaches to the surveillance of HAIs included in the standard HAI-Net ICU protocol of ECDC or the INICC network are the most valuable source of in-depth data enabling descriptions of epidemiological situations, but they are also time consuming and require intensive involvement of staff. Thus, in the case of limited resources, there are light versions of ECDC protocol available. This version was implemented in the hospitals participating in the HAI surveillance programme run by the Polish Society of Hospital Infections, starting from 2014.

The objective of this study was to analyse the occurrence of HAIs in three intensive care units participating in the Polish Society of Hospital Infections (PSHI) surveillance programme in the period 2016–2019, based on the ECDC HAI-Net ICUs recommendations. Incidence rates, clinical forms, and etiological factors of infections in the study group, provided as the results of the analysis, should serve as a benchmark for other hospitals in the country and in Europe and indicate the area of necessary interventions aiming at improving the situation.

## 2. Material and Methods

The hospitals providing data used for this analysis, voluntarily joined the Surveillance of Healthcare-Associated Infections Programme of PSHI, based on recommendations of the HAI-Net ICU ECDC protocol, including the case definitions of clinical forms of infections [19,20]. The light version of protocol was implemented by PSHI for surveillance in intensive care units [21]. The study was conducted in the period 2016–2019 in three hospitals located in southern Poland: Provincial Specialist Hospital of St. Łukasz in Tarnów, Henryk Klimontowicz Specialist Hospital in Gorlice, and Provincial Hospital in Bielsko-Biała. Information on patients with HAI was collected by active surveillance in accordance with the HAI surveillance in ICU protocol methodology [21]. All data entered into the electronic database (Web Program for Active Registration of Hospital Infections, Kraków, Poland) and analysed in this study were previously anonymised. Patients were excluded from the study if their hospital stay in ICU was shorter than 2 days and if their symptoms of infection appeared within 2 days from the start of hospitalization. The following forms of HAI were detected: bloodstream infections (BSI), pneumonia (PN), urinary tract infections (UTI), lower respiratory tract infections (LRI), gastrointestinal infections (GI), skin and soft tissue infections (SST), and systemic infections (SYS).

Cases of pneumonia were divided into subgroups according to the method of microbiological diagnostic used: (PN1) quantitative culture of minimally contaminated material derived from the lower respiratory tract (LRT); (PN2) quantitative culture from a possibly contaminated sample from LRT; (PN4) sputum culture or non-quantitative culture of material from LRT. There were no cases of PN3 or PN5, it is case confirmed by alternative microbiology methods or with no positive microbiology. 

Bloodstream infections were divided into secondary (BSI-S), of unknown origin (BSI-UNK), and associated with central catheter (BSI-CRI). BSI-CRI infections were divided into local (CRI1), systemic (CRI2), and confirmed by microbiological testing (CRI3). BSIs were qualified as secondary (BSI-S) if the same microorganisms were cultured from blood and from another site of infection in the microbiological test. BSIs were classified as: secondary to pneumonia (BSI-PUL), secondary to urinary tract infection (BSI-UTI) and secondary to surgical site infection (BSI-SSI).

Urinary tract infections (UTI) were divided into those with microbiological confirmation (UTI-A) and without microbiological confirmation (UTI-B).

The clinical forms of infections were analysed with the use of epidemiological measures such as: incidence rate = number of HAIs/number of hospitalizations × 100; incidence (density) = number of HAIs/number of hospitalizations × 1000); mortality = number of deaths/number of HAIs × 100.

Statistical analysis calculations included: frequency, percent, mean, and confidence intervals for the mean (95Cl). The differences between the variables were verified with Pearson’s *χ*^2^ test of independence for qualitative variables and ANOVA for quantitative variables, assuming the significance level of *p* < 0.05. The calculations were performed using SPSS 25, IBM SPSS STATISTICS (SPSS—Statistical Package for the Social Sciences STATISTICS 24, Armonk, NY, USA) and Microsoft Excel (Microsoft, Redmond, WA, USA).

The use of the data has been approved by the Bioethics Committee of the Jagiellonian University Medical College (No. KBET/122.6120.118.2016 from 25.05.2016).

## 3. Results

The study included 3028 patients hospitalized for 26,558 person-days (pds) in intensive care units (ICU) from 2016 to 2019. A total of 540 healthcare-associated infections (HAIs) were detected; the incidence per 100 hospitalizations was 17.8%, while the incidence density per 1000 pds was 20.3. The average length of hospital stay in ICU for a patient with HAI amounted to 48 days. The mean age of patients who developed an HAI was 59 years. HAI was diagnosed (on average) on the 25th day from admission to the ICU. Mortality of patients with an HAI was 16%. The infections had various clinical forms. The most numerous group was patients diagnosed with BSI: 209 (incidence rate 6.9%). Patients who developed PN were second most common: 131 (incidence rate 4.3%). UTI was confirmed in 110 patients (incidence rate 3.6%). These were followed by: LRI 43 (incidence rate 1.4%), *Clostridioides difficile* infections (GI-CDI): 25 cases (CDI/100 = 0.8, CDI/1000 = 0.9, CDI/10,000 = 9.4), SST 15 (incidence rate 0.5%) and SYS 7 (incidence rate 0.2%) (Table 1). 

The specified forms of HAIs were analysed through the assessment of patients’ sex, age, average length of stay in the ICU and the onset of infection, duration of infection treatment and mortality. The lowest average age, 53 years, was recorded for SYS infections, and the highest, 68 years, for SST. The effect of the time elapsed from admission into ICU to the onset of HAI was visible. SYS, PN, LRI, and GI-CDI infections appeared less than three weeks after admission, on average, while BSI and UTI developed, on average, after a month (*p* < 0.05). When analysing patient deaths, a large variation concerning individual forms of HAI was observed; infections revealing the greatest mortality were GI 7 (28%), followed by LRI 21 (21.4%), PN 24 (18.2%), BSI 36 (17.2%), UTI 9 (8.2%), and SST 1 (6.7%) (*p* < 0.05). Men dominated among the patients treated (*p* = 0.688) (Table 1).

Analysis of BSI revealed that the majority of infections were of unknown origin BSI-UNK (89 cases, incidence 2.9%, incidence density 3.4/1000 pds), followed by catheter-related bloodstream infections BSI-CRI (64 cases, incidence 2.1%, incidence density 2.4/1000 pds) and secondary bloodstream infections BSI-S (56 cases, incidence 1.8%, incidence density 2.1/1000 pds). Subsequently, 64 cases of catheter-related bloodstream infections (BSI-CRI) were analysed, divided into: CRI1 (8 cases, incidence 0.3%, incidence density 0.3/1000 pds); CRI2 (10 cases, incidence 0.3%, incidence density 0.4/1000 pds); CRI3 (46 cases, incidence 1.5%, incidence density 1.7/1000 pds). Secondary bloodstream infections BSI-S were also analysed in detail: BSI-PUL (20 cases, incidence 0.7%, incidence density 0.8/1000 pds); BSI-UTI (30 cases, incidence 1.0%, incidence density 1.1/1000 pds); BSI-SSI (6 cases, incidence 0.2%, incidence density 0.2/1000 pds). The highest mortality concerned BSI-S: 14 cases (25%), BSI-UNK: 15 (16.9%), and BSI-CRI: 7 (10.9%), *p* = 0.125 (Table 2).

A total of 131 cases of pneumonia (PN) were identified, including: PN1 detected in 58 cases, PN2 in 57 instances, and PN4—16 (types PN3 and PN5 were not found). Mechanical ventilation was conducted in 100% of patients diagnosed with PN1, 87.7% with PN2, and 56.3% with PN4, (*p* < 0.05). Mortality as regards particular forms of PN was also analysed: in PN4: 37.5%, in PN1: 19% and in PN2: 12.3% (*p* = 0.069) (Table 3).

The analysis also included urinary tract infections (UTI). A division was made into UTI–A and UTI–B. In both groups, the majority of patients had a urinary catheter: UTI-A 97 (97%), UTI-B 9 (90%). Mortality in UTI-A was 8%, and in UTI-B it was 10% (*p* = 0.059) (Table 4).

A general analysis of etiological agents of HAI showed that the most frequently isolated bacteria were *Klebsiella pneumoniae* (16.3%), *Acinetobacter baumannii* (14.4%), *Staphylococcus aureus* (11.8%), and *Escherichia coli* (11.4%).

In BSI, the dominant etiological agents were coagulase-negative *Staphylococcus* CNS (19.6%), *K. pneumoniae* (16.7%), and *S. aureus* (14.2%).

PN and LRI were most commonly caused by *A. baumannii* (34.9%), *Staphylococcus aureus* and *Klebsiella pneumoniae* (14.0% each), and *Escherichia coli* (7.7%).

For UTI, the agents were *Escherichia coli* (29.0%), *Klebsiella pneumoniae* (20.2%), and *Enterococcus faecalis* (14.5%).

In SST, the most frequently isolated microorganism was *S. aureus* (43.0%).

The sole pathogen responsible for GI infections was *Clostridioides difficile* (CDI)—25 cases (100%) (Table 5).

## 4. Discussion

In the present study, the incidence of HAI per 100 hospitalizations amounted to 18%. However, analysis of the Polish literature from earlier years presented below demonstrates that the incidence of HAI in Polish ICUs was higher. In a Polish single-centre study by Wieder-Huszla [6], the incidence was 24% (in 2010). An identical incidence of 24% was obtained by Kübler et al. [11] in 2012. Additionally, Kołpa et al. [12], in a Polish single-centre study carried out in southern Poland, recorded an incidence of 28% in 2018. In another study, conducted in 11 ICU units in northern Poland in 2018, the incidence of HAI amounted to 32% [22]. Due to the low availability of Polish HAI studies concerning ICU, our results were also compared to the prevalence rates obtained in the point prevalence survey (PPS) programme [23], according to which, the prevalence in Polish ICUs was 35% in 2011–2012 [23]. In multicentre studies conducted by ECDC, the mean incidence of HAI in ICU was lower and amounted to 8% [24,25].

In this study, the mortality rate in ICU due to HAI was 16%. In 2018, Dubiel et al. [22] demonstrated a significantly higher mortality rate among patients with HAI in Polish ICUs, which equalled 33%.

There is little data on the time elapsed from the admission to the ICU to the diagnosis of HAI. In our own studies, this time was 25 days, on average. Very similar data are found in another Polish 10-year study, where the author gives the average time of 23 days [8].

In this study, the highest incidence was recorded in BSI (7%). In an earlier Polish multicentre study conducted under the HAI-Net surveillance, in 2013–2015, the incidence rate of BSI was identical, i.e., 7% [14]. In a single-centre study conducted by Kołpa et al. [8], the obtained incidence of BSI amounted to 9%. In a multicentre study conducted in the north of Poland in 2018, the incidence of BSI in ICU was 10% [22]. According to the data from ECDC reports, the incidence of BSI in ICUs in European countries was 4%, on average [9,16,24]. There were large discrepancies between the countries participating in the European research; the lowest incidence, of 2%, was recorded in Lithuania and Luxembourg, and the highest, of 5%, in Portugal and Slovakia [9]. There is a visible difference between the European results and the data obtained in our study for BSI—the incidence rate of BSI was almost two-fold lower in European countries. The high incidence rate of BSI in our study is an incentive to take action against infections and to carefully look for the reasons for this state of affairs.

Secondary bloodstream infections (BSI-S) accounted for 27% of all BSIs in our study. In another Polish study, this percentage was much higher, i.e., 47% [21]. ECDC reports revealed that the average percentage of BSI-S in European countries amounted to 34–35% [9,16]. 

The results of our study draw attention to the high number of bloodstream infections originating from the urinary tract (urosepsis; 53%). Wałaszek et al. [25], in a multicentre study conducted in southern Poland, obtained a similar result for bloodstream infections originating from the urinary tract, i.e., 55%. In European studies, these infections (BSI-S-UTI) account for 13% to 19% [9,16] of all BSI-S. The very high percentage that was found in our study may indicate that UTIs are diagnosed too late in the examined ICUs. The data in the present study demonstrate that 36% of bloodstream infections originated from PN. In the study by Dubiel et al. [22], bloodstream infections with a source in PN constituted 19%.

The results of our study also underline a high percentage of BSI with an unknown source of infection, i.e., 43% of all BSI. Corresponding European data are at 21% [16]. The difference is over two-fold, which may indicate deficient diagnostics of BSI in Poland.

In our study, the incidence of PN amounted to 4%. However, analysis of the Polish literature concerning ICUs revealed incidence rates from 6 to 10% [12,26,27]. In a study by Dubiel et al. [10], the incidence of PN in 11 ICUs in northern Poland amounted to 17%. The incidence rate of PN in European countries according to the data from the ECDC report for 2014 amounted to 6% [16,24]. In our study, the mortality rate due to PN was 18%. The results of another multicentre study carried out from 2013 to 2015 in Polish ICUs demonstrate a mortality rate of 11% due to PN [14].

The results of the present study show that UTI is the third most frequent HAI in ICU, because its incidence was 4%. This result was consistent with the incidence of UTI (4%) in an earlier Polish multicentre study in ICU in 2013–2015 [14]. Similarly, in a study by Dubiel et al. [10], the incidence of UTI in 11 units in northern Poland was 4%. European data from ECDC from 2014 quote the incidence of UTI at 3% [20]. The next report from ECDC from 2017 cites a lower incidence of UTI, i.e., 2% [16].

To sum up all types of HAI in our study, the predominant bacteria were *K. pneumoniae* (16.3%), *A. baumannii* (14.4%), and *S. aureus* (11.8%). Similar proportions of these agents were present in a study conducted in Italy among 773 ICU patients with HAI, in which the following were detected: *K. pneumoniae* (20.7%), *A. baumannii* (17.2%), *P. aeruginosa* (13.4%) and *S. aureus* (5.4%) [14].

The most common pathogens responsible for BSI in our study included: coagulase-negative staphylococci (20%), *K. pneumoniae* (17%), and *S. aureus* (14%). Coagulase-negative staphylococci are the microorganisms most frequently isolated from BSI infections found in this study, similarly to the results of Duszyńska et al., where central line-associated bloodstream infections were examined (45%) [13]. In the ECDC report from 2017 [11], the authors also point to coagulase-negative staphylococci as the dominant flora causing BSI (24%). However, Dubiel et al. [22] reported *P. aeruginosa* as the most frequent agents of BSI (25.9%), followed by *K. pneumoniae* (22.0%), and *S.* *aureus* (9.6%). The ECDC-ICU report from 2019 mentions other microorganisms as most often detected in BSI, i.e., the dominance is given to Gram-positive bacteria, such as coagulase-negative staphylococci (23.6%), Enterococcus spp. (14.9%), and *K. pneumoniae* (12.4%) [16].

However, the high proportion of *K. pneumoniae* among the isolates derived from blood in our study is probably associated with the detected secondary bloodstream infections. *K. pneumoniae* is often isolated from urinary tract infections, lower respiratory tract infections and intra-abdominal infections, and bloodstream infections, and can spread rapidly from patient to patient and through the hands of hospital staff, leading to hospital outbreaks. An increase has been observed in the resistance of *K*. *pneumoniae* to many antimicrobials, including to carbapenems.

The most common etiological agent of PN in our study was *A. baumannii* (35%), which was also the dominant microorganism responsible for PN in other Polish studies conducted in southern Poland [12,13,28]. However, Dubiel et al. [22] in their study conducted in northern Poland, reported *P. aeruginosa* as the most frequent with *A. baumannii*. The differences regarding the detected etiological agents of PN can be explained by the spread of endemic *A. baumannii* in southern Poland and in the countries of southern and south-eastern Europe [29].

The microorganisms most commonly isolated from UTI in our study included *E. coli* (29%). In ECDC reports from 2014 and 2017, the most frequently isolated microorganism was also *E. coli* [16,24]. One of the Polish studies conducted in ICU by Duszyńska et al. [30] demonstrated the dominance of *Enterococcus* spp.

*C. difficile* infection (CDI) drew attention in our results. A total of 25 cases were recorded, which puts the incidence at 9/10,000 pds. A study of a network of hospital laboratories in Poland aimed at determining the annual CDI incidence rate in 2011–2013 showed an average incidence of 6/10,000 pds (2011), 9/10,000 pds (2012), and 10/10,000 pds (2013) [31]. A multicentre Canadian study in 2011–2016 established that the incidence density ranged from 2 to 7/10,000 pds [32]. In the ECDC report for 2016, the incidence density in European countries is given as 2/10,000 pds; however, there is a large discrepancy between individual countries, from 2/10,000 pds in Austria to 13/10,000 pds in Estonia. According to the ECDC report [33], the Polish incidence density of CDI amounted to 6/10,000 pds. In our study, mortality due to CDI was 28%. Higher mortality, i.e., 34%, attributable to CDI acquired during hospitalization in ICU, was reported in a single-centre study in the United Kingdom [34]. According to the ECDC report, in European countries, mortality among patients with CDI in 2016 amounted to 21% [33].

As was stated in the Material and Methods section, our study was based on the light version of ECDC HAI-Net ICU protocol. This approach allowed us to draw on only basic epidemiological data, such as incidence of given forms of infections, mortality, and etiological factors, and indirectly some data of the effectiveness of microbiological diagnostics (based on definitions including data on microbiological tests). The surveillance of HAIs based on identification of patients at higher risk and comprising all patients’ data, including those without infections, is highly desirable and valuable. The examples can be detailed analyses of device-associated infections in Polish ICUs presented by Duszyńsa et al. [13] or cluster analyses from the SPIN-UTI Network described by Barchitta et al. [35]. Additionally, not only the outcome measures should be monitored and analysed, but also the process measures, expressing compliance of preventive procedures, especially care bundles in ICUs [13,18]. The most comprehensive approach of infection control and prevention should also take into account data on antibiotic consumption and antimicrobial resistance. All these factors are included in the point prevalence study of ECDC across Europe and in Poland [23,35,36]. However, active, continuous, targeted surveillance of HAIs in ICUs is what ECDC strongly recommends in addition to the point prevalence study. Our results indicate that even surveillance based on the light protocol may provide valuable data about the state and needs of infection control.

## 5. Conclusions

When we compare the epidemiological rates of HAI in our study with data from other European multicentre studies, there is a visible difference concerning the incidence of HAI, to the detriment of our patients. It seems especially important to take measures to reduce bloodstream infections. In view of the high percentage of BSI-UNK, i.e., 43%, it appears necessary to intensify microbiological diagnostics. HAIs caused by *K. pneumoniae* and *A. baumannii* are becoming a challenge. Given the increasing resistance to antimicrobial drugs and the associated therapeutic difficulties, each pathogen which is isolated should be thoroughly analysed for antibiotic susceptibility, and it is also advisable to conduct genetic typing. An alarming aspect of HAI prophylaxis is also CDI and the high mortality associated with it, i.e., 28%.

The implementation of prophylaxis based on reliable analyses of the local epidemiological situation may result in a decrease in the number of HAIs, and thus, also a reduction in mortality and treatment costs.

## Figures and Tables

**Table 1:** The type, number, and incidence of healthcare-associated infections (HAI), patient sex and age, number of days until the onset of HAI, length of hospital stay, mortality in ICU in 2016–2019.

HAI Type	BSI	PN	UTI	LRI	GI-CDI *	SST	SYS	Total	*p*-Value
Number of HAI n (%)	209 (38.7%)	131 (24.2)	110 (20.3)	43 (7.9)	25 (4.6)	15 (2.7)	7 (1.2)	540	n/a
Incidence	6.9	4.3	3.6	1.4	0.8	0.5	0.2	17.8	n/a
Incidence density (per 1000 pds)	7.9	4.9	4.1	1.6	0.9	0.6	0.3	20.3	n/a
Patient age (years) Mean (95% CI)	61 (58–63)	61 (59–64)	54 (50–58)	55 (49–62)	62 (53–70)	68 (61–75)	53 (35–72)	59 (58–61)	<0.05
Days of ICU stay Mean (95% CI)	56 (46–65)	40 (35–45)	52 (41–63)	34 (29–40)	35 (28–41)	48 (38–57)	37 (25–49)	48 (43–52)	0.062
Days until HAI onset Mean (95% CI)	31 (23–38)	19 (15–23)	31 (21–41)	14 (11–18)	13 (9–17)	27 (18–35)	13 (6–19)	25 (22–29)	<0.05
Death n (%)	36 (17.2)	24 (18.2)	9 (8.2)	9 (21.4)	7 (28.0)	1 (6.7)	0 (0.0)	86 (15.9)	<0.05
Patient sex									
Female n (%)	77 (36.8)	43 (31.8)	42 (38.2)	10 (23.8)	8 (32.0)	4 (26.7)	3 (42.9)	186 (34.4)	0.688
Male n (%)	132 (63.2)	89 (68.2)	68 (61.8)	33 (76.2)	17 (68.0)	11 (73.3)	4 (57.1)	354 (65.5)	

Number of patients = 3028, Number of patient-days of stay = 26,558, *Clostridioides difficile* (CDI) 25 cases *(CDI/100 admissions = 0.8; CDI/1000 = 0.9; CDI/10,000 = 9.4), 95%CI—95% confidence intervals for the mean, number (n), not applicable (n/a), healthcare-associated infections (HAI), bloodstream infection (BSI), pneumonia (PN), urinary tract infection (UTI), lower respiratory tract infection (LRI), gastrointestinal infection (GI) *Clostridioides difficile* infection (CDI), skin and soft tissue (SST), systemic infection (SYS).

**Table 2:** Clinical forms of BSI, frequency of occurrence, incidence, incidence density, presence of a central catheter, mortality in ICUs in 2016–2019.

Factor Category	BSI-UNK	BSI-CR			BSI-S			Total	*p*
Number of BSI (n/%)	89 (42.6)	64 (30.6)			56 (26.8)			209 (100.0)	n/a
Incidence (%)	2.9	2.1			1.8			6.9	n/a
Incidence Density/1000 pds	3.4	2.4			2.1			7.9	n/a
Death (n/%)	15 (16.9)	7 (10.9)			14 (25.0)			36 (17.2)	0.130
Presence of a central catheter (CVC)									
No (n/%)	53 (60.7)	0 (0.0)			20 (35.7)			73 (34.9)	<0.01
Yes (n/%)	36 (40.4)	64 (100.0)			36 (64.3)			136 (65.1)	
Type of BSI									
Type of BSI	n/a	CRI1	CRI2	CRI3	BSI-PUL	BSI-UTI	BSI-SSI	n/a	n/a
Number of BSI	n/a	8	10	46	20	30	6	120	n/a
Incidence (%)	n/a	0.3	0.3	1.5	0.7	1.0	0.2	n/a	n/a
Incidence Density/1000 pds	n/a	0.3	0.4	1.7	0.8	1.1	0.2	n/a	n/a

Number (n), 95%CI—95% confidence intervals for the mean, person-days of hospitalization (pds), incidence density per 1000 pds, not applicable (n/a), secondary bloodstream infection (BSI), unknown bloodstream infection (BSI-UNK), catheter-related bloodstream infection (BSI-CRI), local CVC-related no positive blood culture (CRI 1), general CVC-related infection—no positive blood culture (CRI 2), microbiologically confirmed CVC-related bloodstream (CRI 3), bloodstream infection secondary to urinary tract infection (BSI -UTI), bloodstream infection secondary to pneumonia (BSI -PUL ), bloodstream infection secondary to surgical site infection (BSI -SSI).

**Table 3:** Analysis of PN considering the type of microbiological diagnostics and mechanical ventilation, mortality in ICU in 2016–2019.

Factor Category	PN 1	PN 2	PN 4	Total	*p*-Value
Number of PN (n/%)	58 (44.3%)	57 (43.5)	16 (12.2)	131 (100.0)	n/a
Death (n/%)	11 (19.0)	7 (12.3)	6 (37.5)	24 (100.0)	0.069
	Mechanical ventilation				
No (n/%)	0 (0.0)	7 (12.3)	7 (43.8)	14 (10.7)	<0.05
Yes (n/%)	58 (100.0)	50 (87.7)	9 (56.3)	117 (89.3)	

Number (n), not applicable (n/a), 95%CI—95% confidence intervals for the mean, pneumonia diagnosed on the basis of bacteriological diagnostic performed by positive quantitative culture from minimally contaminated lower respiratory tract specimen (PN 1), pneumonia diagnosed on the basis of bacteriological diagnostic performed by positive quantitative culture from possibly contaminated lower respiratory tract specimen (PN 2), pneumonia diagnosed on the basis of positive sputum culture or non-quantitative lower respiratory tract specimen culture (PN 4).

**Table 4:** Analysis of UTI considering the presence of a catheter, mortality in ICU in 2016–2019.

Factor Category	UTI-A	UTI-B	Total	*p*-Value
Number of UTI (n/%)	100 (90.9)	10 (9.1)	110 (100.0)	n/a
Death (n/%)	8 (8.0)	1 (10.0)	9 (100.0)	0.059
	Urinary catheter			
No (n/%)	3 (3.0)	1 (10.0)	4 (3.6)	0.313
Yes (n/%)	97 (97.0)	9 (90.0)	106 (96.4)	

Number (n), not applicable (n/a), 95%CI—95% confidence intervals for the mean, microbiologically confirmed symptomatic urinary tract infection (UTI-A), not microbiologically confirmed symptomatic urinary tract infection (UTI-B).

**Table 5:** Microorganisms causing HAI isolated in microbiological testing, clinical forms of HAI in ICU in 2016–2019.

Microorganism	BSI	PN + LRI	SST	UTI	Total n (%)
**Gram-positive cocci n (%)**	95 (46.6)	27 (18.9)	7 (50.0)	19 (18.2)	148 (31.8)
*Staphylococcus aureus*	29 (14.2)	20 (14.0)	6 (42.8)	0 (0.0)	55 (11.8)
Coagulase-negative staphylococci	40 (19.6)	1 (0.7)	1 (7.1)	3 (2.9)	45 (9.7)
*Enterococcus faecalis*	17 (8.3)	1 (0.7)	0 (0.0)	15 (14.5)	33 (7.1)
Other Gram-positive cocci	9 (4.4)	5 (3.5)	0 (0.0)	1 (1.0)	15 (3.2)
**Enterobacteriales n (%)**	63 (30.8)	46 (32.2)	4 (28.6)	58 (55.8)	171 (36.8)
*Klebsiella pneumoniae*	34 (16.7)	20 (14.0)	1 (7.1)	21 (20.2)	76 (16.3)
*Escherichia coli*	10 (4.9)	11 (7.7)	1 (7.1)	31 (29.0)	53 (11.4)
*Enterobacter cloacae*	14 (6.9)	6 (4.2)	1 (7.1)	4 (3.9)	25 (5.4)
Other *Enterobacteriaceae*	5 (2.4)	9 (6.3)	1 (7.1)	2 (1.9)	17 (3.6)
**Non-fermenting Gram-negative bacteria n(%)**	33 (16.2)	65 (45.4)	3 (21.4)	21 (20.2)	122 (26.2)
*Acinetobacter baumannii*	14 (6.9)	50 (34.9)	1 (7.1)	2 (2.0)	67 (14.4)
*Pseudomonas aeruginosa*	10 (4.9)	9 (6.3)	1 (7.1)	12 (11.5)	32 (6.9)
Other non-fermenting Gram-negative bacteria	9 (4.4)	6 (4.2)	1 (7.1)	7 (6.7)	23 (4.9)
**Others n(%)**	13 (6.4)	5 (3.5)	0 (0.0)	6 (5.8)	24 (5.2)
*Candida* spp.	13 (6.4)	5 (3.5)	0 (0.0)	6 (5.8)	24 (5.2)
**Total**	204 (100.0)	143 (100.0)	14 (100.0)	104 (100.0)	465 (100.0)
*Clostridioides difficile* 25 cases					

Number (n), bloodstream infection (BSI), pneumonia (PN), lower respiratory tract infection (LRI), skin and soft tissue (SST), urinary tract infection (UTI).

## Data Availability

The data presented in this study are available on the reasonable request from the corresponding author. The data are not publicly available due to the fact that the database was made available to the authors primarily for the purpose of their analysis and publication of the results in the form of collective studies.

## Abbreviations

Bloodstream infection (BSI), catheter-related bloodstream infection (BSI-CR), secondary bloodstream infection (BSI-S), bloodstream infection secondary to pneumonia (BSI-PUL), bloodstream infection secondary to surgical site infection (BSI-SSI), bloodstream infection of unknown origin (BSI-UNK), bloodstream infection secondary to urinary tract infection (BSI-UTI), *Clostridioides difficile* infection (CDI), catheter-related infection (CRI), local CVC-related infection, no positive blood culture (CRI1-CVC), general CVC-related infection, no positive blood culture (CRI2-CVC), microbiologically confirmed CVC-related bloodstream infection (CRI3-CVC), central venous catheter (CVC), European Centre for Disease Prevention and Control (ECDC), gastrointestinal infection (GI), healthcare-associated infection (HAI), Healthcare-Associated Infections Surveillance Network (HAI–Net), intensive care unit (ICU), lower respiratory tract infection (LRI), person-days of hospitalization (pds), pneumonia (PN), bacteriological diagnostic performed by positive quantitative culture from minimally contaminated lower respiratory tract specimen (PN1), bacteriological diagnostic performed by positive quantitative culture from possibly contaminated lower respiratory tract specimen (PN2), alternative microbiological methods (PN3), pneumonia based on positive sputum culture or non-quantitative lower respiratory tract specimen culture (PN4), no positive microbiology (PN5), skin and soft tissue (SST), systemic infection (SYS), urinary tract infection (UTI), microbiologically confirmed symptomatic urinary tract infection (UTI-A), not microbiologically confirmed symptomatic urinary tract infection (UTI-B).
